# Diagnostic relevance of spatial orientation for vascular dementia: A case study

**DOI:** 10.1590/1980-57642018dn12-010013

**Published:** 2018

**Authors:** Gillian Coughlan, Emma Flanagan, Stephen Jeffs, Maxime Bertoux, Hugo Spiers, Eneida Mioshi, Michael Hornberger

**Affiliations:** 1 Norwich Medical School, University of East Anglia, Norwich, UK; 2 Institute of Behavioural Neuroscience, Department of Experimental Psychology, University College London, London, UK; 3 School of Health Sciences, University of East Anglia, Norwich, UK; 4 Dementia and Complexity in Later Life, Norfolk and Suffolk NHS Foundation Trust, Norwich, UK

**Keywords:** spatial orientation, egocentric, allocentric, vascular dementia, VaD, executive function, neurodegeneration, orientação espacial, orientação egocêntrica, orientação alocêntrica, demência vascular, função executiva, neurodegeneração

## Abstract

Spatial orientation is emerging as an early and reliable cognitive biomarker of Alzheimer’s disease (AD) pathophysiology. However, no evidence exists as to whether spatial orientation is also affected in vascular dementia (VaD).

**Objective:**

To examine allocentric (map-based) and egocentric (viewpoint-based) spatial orientation in an early stage VaD case.

**Methods:**

A spatial test battery was administered following clinical and neuropsychological cognitive evaluation.

**Results:**

Despite the patient’s complaints, little evidence of episodic memory deficits were detected when cueing was provided to overcome executive dysfunction. Similarly, medial temporal lobe-mediated allocentric orientation was intact. By contrast, medial parietal-mediated egocentric orientation was impaired, despite normal performance on standard visuospatial tasks.

**Conclusion:**

To our knowledge, this is the first in-depth investigation of spatial orientation deficits in VaD. Isolated egocentric deficits were observed. This differs from AD orientation deficits which encompass both allocentric and egocentric orientation deficits. A combination of egocentric orientation and executive function tests could serve as a promising cognitive marker for VaD pathophysiology.

## RESUMO

A orientação espacial está emergindo como um biomarcador cognitivo precoce e confiável da fisiopatologia da doença de Alzheimer (DA). No entanto, não existe evidência de que a orientação espacial também seja afetada na demência vascular (DVa).

**Objetivo:**

Examinar a orientação espacial alocêntrica (baseada em mapas) e egocêntrica (baseada no ponto de vista) em um caso de DVa em fase incial.

**Métodos:**

Uma bateria de testes espaciais foi administrada após avaliação clínica e neuropsicológica cognitiva.

**Resultados:**

Apesar das queixas do paciente, poucas evidências de déficits de memória episódica foram detectadas quando foram fornecidas pistas para superar a disfunção executiva. Da mesma forma, a orientação alocêntrica mediada pelo lobo temporal medial estava intacta. Em contrapartida, a orientação egocêntrica mediada pela região parietal medial estava comprometida, apesar do desempenho normal em tarefas visuoespaciais padrão.

**Conclusão:**

Pelo nosso conhecimento, esta é a primeira investigação aprofundada dos déficits de orientação espacial na DVa. Foram observados déficits egocêntricos isolados. Isso difere dos déficits de orientação da DA que abrangem déficits de orientação alocêntricos e egocêntricos. Uma combinação de orientação egocêntrica e testes de função executiva poderia servir como um marcador cognitivo promissor para a fisiopatologia de DVa.

Deficits in spatial orientation are an emerging early marker for Alzheimer’s disease (AD) pathophysiology.^1^^-^ 5 They have been strongly linked to medial temporal and intra-parietal regional changes in incipient and present AD pathophysiology.^6^^,^ 7 However, at this stage it is not clear if vascular dementia patients also display any spatial orientation deficits. Such a distinction is important as vascular dementia (VaD) is the second most common form of dementia and the diagnostic differentiation of both dementias is challenging with patients commonly complaining of generic memory complaints.^8^^,^ 9 Importantly, VaD patients often show intact medial temporal lobe function, while frontal and parietal regions are compromised due to white matter lesions in the superior frontal fasciculus.^10^^,^ 11 Therefore, apparent memory problems in VaD are more likely due to frontal executive and parietal visuospatial deficits than medial temporal memory mediated processes. In the current case study, we explored whether spatial orientation performance could help detect VaD and generate a different profile to AD. We hypothesised that if the case shows spatial orientation deficits, these should be limited to egocentric parietal orientation problems but that allocentric medial temporal processes should remain intact.

## PARTICIPANT

We report the case of RK, a 65-year-old married man, with six years of secondary education, who worked as a truck driver and window cleaner. A diagnosis of VaD was made in March 2017, he then presented at our dementia research clinic with memory complaints. He reported a short history of behavioural and psychological symptoms including apathy, depression and agitation/aggression. His medical history also revealed hypercholestrol, stage 2 hypertension, a BMI of 30 and life-long cigarette smoking. There was a strong family history of hypercholestrol (both parents and siblings) and heart disease-related death in both parents.

### Procedures

RK underwent clinical and cognitive assessments, including neuropsychological assessments (Table 1). Both RK and his carer reported memory problems, such as misplacing keys and forgetting appointment. These issues are most likely due to attentional and executive demands, as recent family events were recalled without difficulty. Problems related to executive function, such as misplacing medication and poor finance management, were also reported. Importantly, spatial orientation difficulties were a central concern for both RK and the carer, and included complaints of disorientation on previously familiar routes and when using public transport, which had led to significant safeguarding concerns by the family. Based on these concerns, an additional spatial test battery was administered.

**Table 1:** Physical and neuropsychological background.

**Age**	69			
**Nationality**	British			
**Blood pressure**	• Systolic: 165 mmHg (lying), 158 mmHg (standing)			
	• Diastolic: 100 mmHg (lying) 101 mmHg (standing)			
**Heart rate**	55 bpm (lying) 61 bpm (standing)			
**Height**	175 cm			
**Weight**	91 kg			
**Body Mass Index**	30			
**Medication management**	• Clopidogrel (75 mg)	Simvastatin (40 mg)		
	• Losarten potassium (100 mg) High dosage	Bendroflumethiazide (2.5 mg)		
**General Cognitive Ability Test**	Addenbrooke's Cognitive Examination - III (ACE)		**Patient score**	**Control score**
	• ACE attention		18	(17 / 1.9)
	• ACE memory		18	(23 / 2.7)
	• ACE fluency		04*	(12 / 2.0)
	• ACE language		26	(25 / 0.9)
	• ACE visuospatial		16	(14 / 1.0)
	• ACE total		82	(92 / 4.7)
**Visuospatial functioning**	Visual Object and Space Perception Battery (VOSP)			
	• Dot counting		09/10	
	• Position		20/20	
	• Cube		10 /10	
	Rey Complex Figure (ROCF)			
	• Construction		25*	(33.7 / 1.6)
	• Reconstruction (3-minute delay)		09	(19 / 4.5)
**Episodic Memory**	Free and Cued Selective Reminding Test (FCSRT)			
	• Free immediate recall		15/48*	
	• Cued immediate recall		33/48	
	• Free delayed recall		06/16*	
	• Cued delayed recall		10/16	
**Language Ability**	Sydney Language Battery			
	• Naming		29/30	
	• Comprehension and repetition		10/10	
	• Semantic association		28/30	
**Executive Function / Mental Flexibility**	INECO Frontal Screening Test			
	• Motor series		3/3	
	• Interference sensitively		2/3	
	• Inhibitory control		2/3	
	• Digit backwards		2/6*	
	• Verbal working memory		1/2	
	• Spatial working memory		1/4*	
	• Proverbs		0.5/3*	
	• Hayling test		5/6	
	• Working memory index		3/10*	
	• Total		16.5*	
	Trail Making Task		**Part A**	**Part B**
	• Time (sec)		79	117
	• Errors		0	2
**Social Cognition Mini-SEA**	• Non-Faux-pas		10/10	
	• Faux-pas (ToM)		21/30*	
	• All stories		31/40	
	• Control		19/20	
	• Facial Emotion Recognition		30/35	

* Significant differences. Standard mean score and standard deviation representing an aged-matched control group are in parenthesis. Note control scores were only available for the ACE-III and the ROCF test.

**Figure 1:**
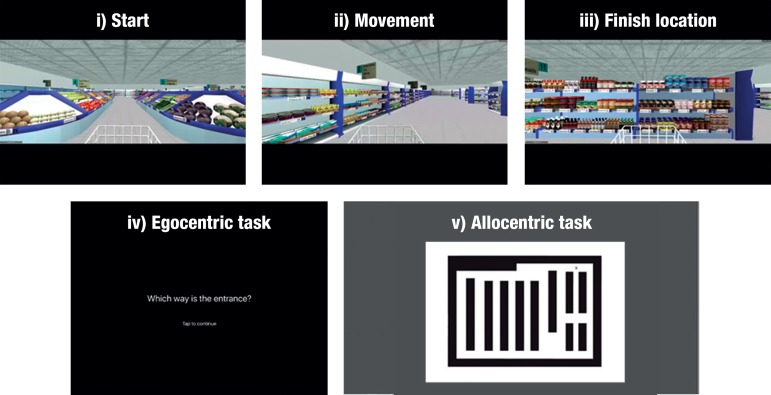
Screenshots from the Supermarket task, showing i) start viewpoint; ii) movement during an example video clip; iii) end location of an example video clip; iv) ‘onscreen instructions prompting the participant to indicate the direction of their starting point’; v) the supermarket map participants use to indicate their finishing location and their heading direction when the video clip ends

The spatial battery consisted of three spatial measures: The Supermarket task, The Statue task and the Clock test. The Supermarket task is an ecologically valid tool adopted to assess the integrity of egocentric and allocentric heading orientation and spatial memory in dementia. Participants are shown short video clips (7 seconds) of a virtual reality supermarket, whereby the person in the video is navigating from the entrance to a finishing location automatically (Figure 1). Once the video clip stops, participants are asked to indicate in real-life the direction of their starting point (egocentric orientation). In a second step, participants are given a map of the Supermarket and are asked to indicate where they are on the map (allocentric orientation) and what direction they are facing in the supermarket (heading orientation). More details can be found here.^1^^,^ 2

**Figure 2:**
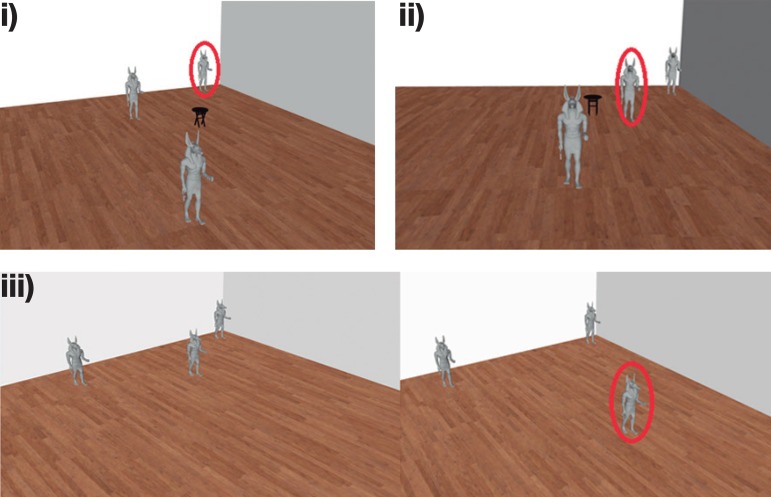
Screenshots from wall, stool and memory subtasks of the Statue test. Participants view images and are asked i) identify the statue closest to the wall (permanent landmark), ii) identify the statue closest to the stool (transient landmark), iii) identify which statue moved its location. Note, red circled figures are only shown for illustration purposes to identify the correct choice for each example, which was not shown to the participants.

The Statue Test requires participants to make spatial judgements for a room with 3 statues and a small stool (Figure 2). Participants are asked to indicate i) the statue closest to one of the walls (permanent landmark); ii) the statue is closest to the stool (transient landmark); iii) which of the three statues moved its location after a delay. Each of these sub-tasks includes an *easy, medium, and hard* condition. The landmark decisions are thought to rely on intra-parietal lobes, whereas the memory condition is typically thought to rely on the medial temporal lobe.

The Clock test requires participants to imagine they are standing in the centre of a large clock facing, e.g., the number 12. Participants are asked to then point in real-life to different numbers on the clock face. For example, “Can you point to the number 9?” (*Answer: pointing left)*. The questions increase in complexity across the test and require medial parietal mediated mental imagery, rotation and egocentric processes, with no episodic memory demand. The study was approved by the UK National Research Ethics Service (NRES: 16/LO/1366).

## RESULTS

### Analysis

We compared the case to a control sample (*N* = 13) with a mean age of 63 (SD = 4.8), who underwent similar testing. RK was contrasted against the controls via a modified paired sample t-test developed by Crawford and colleagues,^12^^,^ 13 resulting in a Z-case-control (z_cc_) score as an interval estimate of the effect size.

### Neuropsychological evaluation (Table 1)

RK achieved a score of 82 on the ACE-III, and cognitive deficits on free recall (immediate and delayed), executive function (spatial working memory, digital backwards, proverbs), social cognition and verbal fluency measures were observed. Importantly, on the FCSRT, his deficits were only present in free recall; once semantic cues were provided, RK could recall all verbal material, indicating executive dysfunction as the main contributor to the episodic memory deficits. Similarly, for visual episodic memory, the planning of the ROCF copy was disorganised due to executive deficits, which resulted in low recall score. RK’s performance on the theory of mind (ToM) subset of the mini-SEA further suggests a partial deficit in social cognition. Importantly, basic visuo-perception and spatial discrimination (VOSP) were in the normal range, indicating no basic visual problems. Language skills were also in the normal range.

### Spatial orientation performance (Table 2)

On the Supermarket task, RK showed significant egocentric navigational impairments (t = -9.529, *p* <.000, z_cc_ = -9.889), i.e. failing to point back to the starting point correctly. Similarly, heading orientation (correct judgement of facing direction after travel period) was also impaired, albeit less severely (t = -2.983, *p* = 0.01, z_cc_ = -3.095). By contrast, allocentric information, i.e. indicating the place location in the supermarket test, was not significantly different from the control group (t = -1.537, *p* >0.05, z_cc_ = -0.206).

**Table 2:** Total scores, standard deviations (SD), Z-case-control (Zcc) scores and confidence intervals (CI) from a modified paired sample t-test for patient and control group on the spatial test battery.

Spatial measures	Condition	Patient score	Control sample mean (N = 13)	(SD)	t-value	p value	Effect size (Z_-CC_)	95% CI
**Statue test**	Wall Easy	4	4	0	0.00	NS	-0.00	-0.544 to 0.544
	**Wall Medium**	**1**	**2.6**	**0.5**	**-3.085**	0.01*	**-3.160**	**-4.511 to -1.789**
	Wall Hard	0	0.3	0.6	-0.000	NS	-0.00	-1.083 to 0.091
	Stool Easy	4	3.7	0.4	-0.723	NS	-0.750	0.119 to 1.357
	**Stool Medium**	**0**	**2.2**	**0.8**	**-2.590**	0.02*	**-2.687**	**-3.869 to -1.484**
	Stool Hard	0	0.3	0.6	-0.482	NS	0.500	-1.069 to 0.088
	Memory Easy	4	3.9	0.2	-0.483	NS	0.500	-0.088 to 1.069
	Memory Medium	2	2.5	0.6	-0.623	NS	0.622	0.525 to 1.563
	Memory Hard	0	0.2	0.7	-0.321	NS	0.333	-0.886 to 0.233
**Supermarket test**	**Egocentric navigation**	**4**	**12.9**	**0.9**	**-9.529**	<0.001**	**-9.889**	**-13.825 to -5.949**
	Allocentric memory	1.5	8.1	3.2	-0.201	NS	-0.206	-3.028 - 1.070
	**Heading Direction**	**6**	**12.5**	**2.1**	**-2.983**	0.01*	**-3.095**	**-4.422 to -1.746**
**The Clock test**	**Cardinal (Verbal Response)**	**1**	**3.9**	**0.9**	**-3.105**	<0.01*	**-3.222**	**-4.596 to -1.829**
	**Right angle (pointing response)**	**1**	**3.6**	**0.6**	**-4.176**	<0.001**	**-4.333**	**-6.120 to -2.532**
	Lateral, behind, (mixed response)	1	3.9	1.7	-1.644	NS	-1.706	-2.558 to - 0.826
	**Total Score**	**3**	**11**	**2.6**	**-2.965**	0.01*	**-3.077**	**-4.398 to -1.736**

Significant differences are market bold. P value representing a two-tailed probability that case score differs from controls.

On the statue task, RK showed no significant differences for performance on the *easy* and *hard* versions of all conditions, due to ceiling and floor effect. However, in the *medium* condition, abnormal scores were detected on both the wall (t = -3.085, *p* = 0.01, z_cc_ = -3.160) and stool (t = -2.590, *p* = 0.02, z_cc_ = -2.687) condition only, showing deficits on visual judgements for permanent and transient objects. RK’s memory performance was comparable to healthy controls.

Finally, the patient’s clock test scores were significantly lower than those of controls (t = -2.965, *p* = 0.01, z_cc_= -3.077) reflecting poor higher visual (mental rotation) and egocentric processing abilities.

## DISCUSSION

To our knowledge, this is the first description of human spatial orientation deficits in a VaD case. As predicted, RK shows a typical neuropsychological profile of VaD that includes executive function impairments, as well as memory deficits indicating frontal lobe dependant executive symptomology.^14^^,^ 15 These deficits are accompanied by hypercholestrol, elevated BMI and stage 2 hypertension.^16^ Normal performance on allocentric orientation measures associated with the medial temporal lobe^17^^-^ 21 corroborate intact episodic memory after cueing. Deficits in egocentric orientation, dependent mainly on the medial parietal cortex,^6^^,^ 22 ^,^ 23 denote a clear and isolated spatial impairment. More specifically, RK performed worse than controls only on the egocentric portions of the spatial tasks. By contrast, standard neuropsychological visuospatial tasks failed to detect these spatial deficits, despite being one of RK’s main symptoms which causing his family significant concern.

Diagnostically, patients with early AD disease usually exhibit both allocentric and egocentric deficits,^1^^,^ 2 ^,^ 5 ^,^ 24 while RK had specific egocentric difficulties. Therefore, detecting only egocentric deficits along with executive function impairments would not only suggest underlying VaD pathophysiology, but may also allow the diagnostic differentiation of AD from VaD. This suggestion needs to be verified in future group and AD comparison studies. Nevertheless, findings reported here form a promising step towards advancing diagnostic tests for VaD, for which cognitive testing is currently very limited and non-specific.^25^^,^ 26 More generally, spatial testing has a promising future as it is highly ecological, resulting in high patient test compliance but also involving very little verbal material. For these reasons, spatial tests are ideal for cross-cultural testing and are potentially less vulnerable to the impact of educational attainment.

Overall, we report a VaD case with selective egocentric spatial orientation deficits, which tap into the medial parietal changes that are typically associated with this condition. Spatial orientation therefore promises to complement executive testing in VaD to detect the underlying disruption of frontoparietal networks.
